# Diagnostic value of metagenomic next-generation sequencing of bronchoalveolar lavage fluid for the diagnosis of suspected pneumonia in immunocompromised patients

**DOI:** 10.1186/s12879-022-07381-8

**Published:** 2022-04-29

**Authors:** Pengcheng Lin, Yi Chen, Shanshan Su, Wengang Nan, Lingping Zhou, Ying Zhou, Yuping Li

**Affiliations:** 1 Department of Pulmonary and Critical Care Medicine, The First Affiliated Hospital of Wenzhou Medical University, South Baixiang, Ouhai, Wenzhou, Zhejiang 325015 People’s Republic of China; 2 Department of Infectious Diseases, The First Affiliated Hospital of Wenzhou Medical University, Wenzhou, 325015 Zhejiang China

**Keywords:** Metagenomic next-generation sequencing, Conventional microbiological tests, Bronchoalveolar lavage fluid, Diagnostic performance, Immunocompromised

## Abstract

**Background:**

To evaluate the diagnostic value of metagenomic next-generation sequencing (mNGS) of bronchoalveolar lavage fluid (BALF) in immunocompromised patients for the diagnosis of suspected pneumonia in comparison with that of conventional microbiological tests (CMTs).

**Methods:**

Sixty-nine immunocompromised patients with suspected pneumonia received both CMTs and mNGS of BALF were analyzed retrospectively. The diagnostic value was compared between CMTs and mNGS, using the clinical composite diagnosis as the reference standard.

**Results:**

Sixty patients were diagnosed of pneumonia including fifty-two patients with identified pathogens and eight patients with probable pathogens. Taking the composite reference standard as a gold standard, 42 pathogens were identified by CMTs including nine bacteria, 17 fungi, 8 virus, 6 *Mycobacterium Tuberculosis*, and two *Legionella* and 19(45%) of which were detected by BALF culture. As for mNGS, it identified 76 pathogens including 20 bacteria, 31 fungi, 14 virus, 5 *Mycobacterium Tuberculosis*, four *Legionella* and two *Chlamydia psittaci*. The overall detection rate of mNGS for pathogens were higher than that of CMTs. However, a comparable diagnostic accuracy of mNGS and CMTs were found for bacterial and viral infections. mNGS exhibited a higher diagnostic accuracy for fungal detection than CMTs (78% vs. 57%, *P* < 0.05), which mainly because of the high sensitivity of mNGS in patients with *Pneumocystis jirovecii* pneumonia (PJP) (100% vs. 28%, *P* < 0.05). Nineteen patients were identified as pulmonary co-infection, mNGS test showed a higher detection rate and broader spectrum for pathogen detection than that of CMTs in co-infection. Moreover, *Pneumocystis jirovecii* was the most common pathogen in co-infection and mNGS have identified much more co-pathogens of PJP than CMTs.

**Conclusions:**

mNGS of BALF improved the microbial detection rate of pathogens and exhibited remarkable advantages in detecting PJP and identifying co-infection in immunocompromised patients.

**Supplementary Information:**

The online version contains supplementary material available at 10.1186/s12879-022-07381-8.

## Background

Pneumonia is the most common cause of morbidity and mortality in immunocompromised patients, for which early identification of causative pathogens is necessary [1]. However, the conventional microbiological tests (CMTs) methods are time consuming and represent a low rate of positive detection, which cannot meet the diagnosis needs of an immunocompromised patient. Even with the combination of culture and culture-independent techniques (nucleic acid amplification testing and serological testing), the etiological diagnosis of severe pneumonia in immunocompromised patient is usually unclear.

Metagenomic next-generation sequencing (mNGS), an unbiased pathogen detection method, has been used in infectious diseases increasingly in recent decades. It is mainly used for assessing the sterile body fluids in the clinical setting, including cerebrospinal fluid, joint effusion and blood [2–4]. For non-sterile body fluids, such as bronchoalveolar lavage fluid (BALF), the applications of mNGS are greatly limited, especially for immunocompromised patients. Pan et al. explored the application of mNGS of BALF in thirteen immunocompromised patients for the diagnosis of pneumonia and showed improved detection of opportunistic pathogens [5]. However, Peng et al. reported a similar diagnostic value of mNGS of BALF in immunocompromised patients to the CMTs for all types of pathogens [6].

The aim of our study was to evaluate the diagnostic performance of mNGS of BALF for pneumonia in immunocompromised patients in comparison with that of CMTs. Moreover, we intended to show diagnostic accuracy rates for different types of pathogens.

This study received approval from the Ethics Committee of the First Affiliated Hospital of Wenzhou Medical University (No. 2020-111) and was conducted in accordance with the Declaration of Helsinki (as revised in 2013). Informed consent to publish the information was obtained from the personal patient.

## Patients and methods

### Patients

From September 2018 to December 2020, all immunocompromised patients with suspected pneumonia admitted to the First Affiliated Hospital of Wenzhou Medical University were retrospectively investigated.

The inclusion criteria of patients were as follows: (A) at least eighteen years old; (B) had an immunocompromised condition; (C) admitted to the Department of Pulmonary and Critical Care Medicine due to suspected pneumonia; (D) had mNGS of BALF within 48 h after admission, and other relevant samples available for the conventional microbiological tests in standard procedures;

Immunocompromised status [7] (≥ 1 of the following risk factors were found): (A) hematologic cancer; (B) chemotherapy during the last three month; (C) chronic steroid (> 0.3 mg/kg/d of prednisone-equivalent for ≥ 3 weeks) or biologic drug use for autoimmune diseases or other immunosuppressive therapy; (D) solid-organ transplant receipt during the last six months; (E) neutropenia; (F) acquired or inherited severe immunodeficiency.

Patients with suspected pneumonia meet the both criteria below: (A) new-onset fever, cough, expectoration or dyspnea; (B) new-onset abnormal chest imaging manifestations.

### Data collection

The following data were recorded: age, sex, underlying diseases and clinical manifestations, illness severity (pneumonia severity index (PSI) score), chest radiology, results of laboratory examination including microbiological testing and patient outcomes (regular clinic follow-up or telephone conversation).

### Microbiological tests

All patients underwent the operation of bronchoalveolar lavage following a standard safety protocol [8]. The BALF specimens were divided into aliquots. One aliquot was set for bacterial and fungal smear (including Gomori methenamine silver (GMS) staining and acid-fast stain) and culture. One aliquot was submitted for the detection of galactomannan antigen and cryptococcus capsular polysaccharide antigen. X-pert MTB/RIF detection of DNA sequences specific for *Mycobacterium Tuberculosis* (MTB) and real-time polymerase chain reaction (PCR) for c*ytomegalovirus* (CMV) and *influenza A/B virus*. The other aliquots (5 mL, settled in a sterile sputum container, stored at − 20 °C) were sent to BGI-Huada Genomics Institute (Shenzhen, China) for detection.

For the other samples, urine was submitted for the detection of antigen of *Legionella pneumophila* and *Streptococcus pneumoniae*. Peripheral blood samples were sent for the detection of the galactomannan antigen to *Aspergillus*, cryptococcus capsular polysaccharide antigen to *Cryptococcus* and immunoglobulin G and M antibodies to *parainfluenza, adenovirus*, *CMV*, *Chlamydia pneumoniae* and *Mycoplasma pneumoniae* using commercial enzyme-linked immunosorbent assays (Ani Labsystems) according to the manufacturer’s instructions.

### mNGS of BALF

The procedure of mNGS for BALF samples consists of nucleic acid extraction, library construction, sequencing and bioinformatic analyses.

### Nucleic acid extraction

A 1.5-mL microcentrifuge tube with 0.6 mL of BALF sample taken from patient, Lyticase (Tiangen, Beijing, China) and 1 g of 0.5 mm glass bead (BioSpec Products, OK, USA) was attached to a horizontal platform on Vortex-Genie2 vortex mixer (Scientific Industries, USA) and agitated vigorously (2800–3200 rpm for 30 min). After agitation, 0.3 mL sample was separated into a new 1.5-mL microcentrifuge tube, and DNA was extracted in 40 μL elution volume by the TIANamp Micro DNA Kit (DP316 Tiangen Biotech, Beijing, China).

### Library construction

DNA libraries (DNA engaged in library preparation was 100–400 ng) were constructed by DNA fragmentation, end repair, adapter-ligation, and PCR amplification using the MGIEasy DNA Library Prep Kit (MGI, Wuhan, China). Agilent 2100 Bioanalyzer Instrument and Qubit 3.0 (Thermo Fisher Scientific, USA) platform were used for library quality control. Quality qualified libraries were sequenced on MGISEQ-2000 platform [9].

### Sequencing and bioinformatic analyses

Initially, low-quality reads were removed for obtaining high-quality sequencing data. Then, human host sequences were recognized and excluded by mapping against the human reference genome (hg19) using Burrows-Wheeler Alignment [10]. Finally, the remaining sequence data by removal of low-complexity reads were aligned to 4 Microbial Genome Databases (including viruses, bacteria, fungi, and parasites), which were downloaded from NCBI (ftp://ftp.ncbi.nlm.nih.gov/genomes/). RefSeq contains 4,945 whole-genome sequences of viral taxa, 6,350 bacterial genomes or scaffolds, 1064 fungi related to human infection, and 234 parasites which were associated with human diseases.

### Criteria for mNGS result

Oral commensals, which were normally parasitic in the human oropharynx and usually without clinically significant, were easily to be detected and always will be carefully considered in combination with a clinical analysis in clinical practice. So, we use a mix of Miao’s [11] and Peng’s [6] diagnostic criteria in our diagnostic criteria, which was more close to clinical practice.

(1) Bacteria (mycobacteria excluded), virus and parasites: A microbe was considered clinically significant microbes (CSMs) when its coverage rate scored tenfold greater than that of any other microbes according to Miao’s study [11]. (2) Fungi: A microbe was considered CSMs when its coverage rate scored fivefold greater than that of any other fungus due to its low biomass in DNA extraction [12, 13]. (3) Mycobacteria: MTB was defined as positive when at least one read was mapped to either the species or genus level because of the low possibility for contamination and difficulty of DNA extraction [14]. Nontuberculous mycobacteria (NTM) were considered when the mapping read number was in the top 10 in the bacteria list because of the low possibility for contamination [15] and low yield rate [16]. Regardless of coverage rate, oral commensals were not defined as CSMs unless they were deemed to be significant by the physicians or proven otherwise [6]. Coverage rate was regarded as the measurement parameter in our study because of the consideration of confounding factors such as pathogen genome size, nucleic acid contamination and total number of sequencing reads [12].

### Clinical composite diagnosis as the reference standard

The medical records such as clinical features, laboratory examination, microbiological tests (including mNGS and CMTs), chest imaging and therapeutic response, were reviewed independently by the two physicians who specialize in the management of infection in immunocompromised hosts to determine whether the patients had infectious etiology or not and identify the pathogens (definite or probable). An in-depth discussion was performed when there was any disagreement between the two physicians and another senior physician was consulted if consensus could not be reached.

### Statistical analysis

SPSS 22.0 (IBM Corporation) was used for analysis. Continuous variables were reported as the mean ± standard deviation (SD) or the median (25th, 75th percentiles) depends on they were normally distributed or non-normal distribution. Categorical variables were presented as numbers (percentages). Determination of microbiological etiology and clinical composite diagnosis were used as the reference standard. The chi-square test, McNemar’s test or Fisher’s exact test was used to compare the diagnostic performance of mNGS and CMTs. Test concordance was assessed using the kappa (κ) statistic. All tests were two-tailed and *P* < 0.05 was considered significant.

## Results

### Patient characteristics

A total of 69 patients including 44 male (64%) were enrolled in this study. The mean age was (57 ± 15) years. Thirty-three patients (48%) received a long-term corticosteroid therapy for solid-organ transplantation or autoimmune diseases and twenty-eight patients (41%) treated with chemotherapy for solid tumors or hematologic malignancy. The median PSI score was (127 ± 51). Twenty-two patients (32%) received invasive mechanical ventilation and 25 of 69 patients developed septic shock. The clinical features of all patients including demographic characteristic, laboratory findings and prognosis were shown in Table 1.

**Table 1:** Characteristic of 69 immunocompromised patients

Characteristic	69 patients
Age (year), mean ± SD	57 ± 15
Gender, male, n (%)	44(64)
Immunocompromised status, n (%)	
Hematologic malignancy	23(33)
Solid-organ transplantation	14(20)
Solid tumour receiving chemotherapy	5(7)
Immunosuppressive therapy^a^	21(30)
Prolonged corticosteroid therapy^b^	33(48)
Disease severity	
PSI score, mean ± SD	127 ± 51
Invasive mechanical ventilation, n (%)	22(32)
Septic shock, n (%)	25(36)
Laboratory findings	
WBC (10^9^/L), median (Q1, Q3)	6.58(4.93,11.05)
Neutrophils (10^9^/L), median (Q1, Q3)	5.66(3.23,9.39)
Lymphocyte count (10^6^/L), median (Q1, Q3)	0.73(0.35,1.17)
PaO_2_/FiO_2_, mean ± SD	196 ± 98
Albumin (g/L), mean ± SD	30 ± 7
Serum creatinine(mmol/L), median (Q1, Q3)	80(57, 127)
LDH (U/L), median (Q1, Q3)	432(304, 726)
CRP (mg/l), median (Q1, Q3)	88(42,149)
PCT (ng/ml), median (Q1, Q3)	0.27(0.11,1.20)
outcome	
Total 30-day mortality, n (%)	14(20)

*PSI* pneumonia severity index, *WBC* white blood cell, *LDH* lactate dehydrogenase, *CRP* C-reactive protein, *PCT* procalcitonin

^a^More than 2 weeks. Underlying diseases include Dermatomyositis (2), Leukemia (1), connective tissue diseases (6), Solid-organ transplantation (9), Hemophilia (1), Chronic nephritic syndrome (1), Thrombocytopenia (1)

^b^Defined as > 0.3 mg/kg/d of prednisone-equivalent for ≥ 3 weeks. Underlying diseases include Dermatomyositis (1), Connective tissue diseases (9), Solid-organ transplantation (10), Hemophilia (1), Nephrotic syndrome (6), Thrombocytopenia (1), interstitial lung disease (1), Hematologic malignancy (2), Intracranial tumour (1), Dermatology (1)

According to the retrospective review of medical records including mNGS and CMTs, 60 patients were diagnosed with pneumonia including 52 patients with definite pathogens and eight patients with probable pathogens. The most common pathogens were *Pneumocystis Jiroveci *(42%), *Cytomegalovirus *(18%) and *Pseudomonas aeruginosa *(12%). Another nine patients were considered non-infection disease including rejection of bone marrow with lung involvement (n = 2), connective tissue diseases (n = 3) or drug (n = 2) related interstitial lung disease, lymphoma with lung involvement (n = 1) and lung cancer (n = 1).

### Pathogen detection by mNGS relative to CMTs

Among 60 patients of confirmed pneumonia, 42 pathogens were identified by CMT and 19 (45%) of which were detected by BALF culture (including nine bacteria, six *Aspergillus*, two *Cryptococcus neoformans* and two *Legionella*). The average pathogen-identification time consumption of culture was 119 ± 87 h. Eight virus were detected by PCR, seven *Pneumocystis Jiroveci* were detected by GMS staining, six MTB were detected by using the GeneXpert TB PCR kit and two cases of cryptococcosis were diagnosed by cryptococcus capsular polysaccharide antigen.

**Fig. 1:**
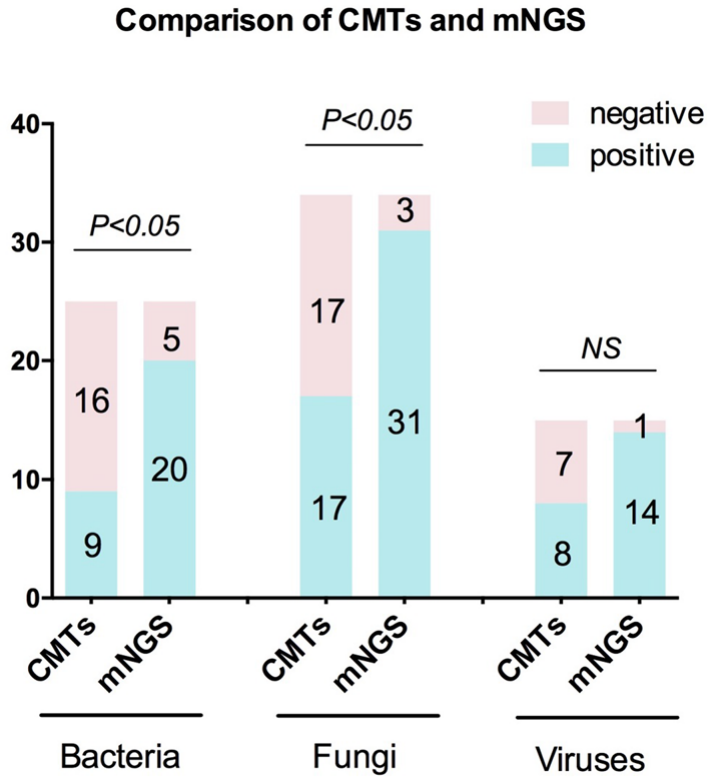
Comparison of CMTs and mNGS test for the different classes of pathogens. The number of positive samples (y-axis) for pairwise mNGS and CMTs is plotted against the bacteria, fungus and virus groups (x-axis). *mNGS* metagenomic next-generation sequencing; *CMTs* conventional microbiological tests

As for mNGS, it identified 76 pathogens (including 20 bacteria, 31 fungi, 14 virus, five MTB, four *Legionella* and two *Chlamydia psittaci*). The turnaround time of mNGS process was 24 to 48 h. The comparison of different classes of pathogens detected by the CMTs and mNGS was shown in Fig. 1.

### Comparison of mNGS and CMTs pathogen detection methods

**Fig. 2:**
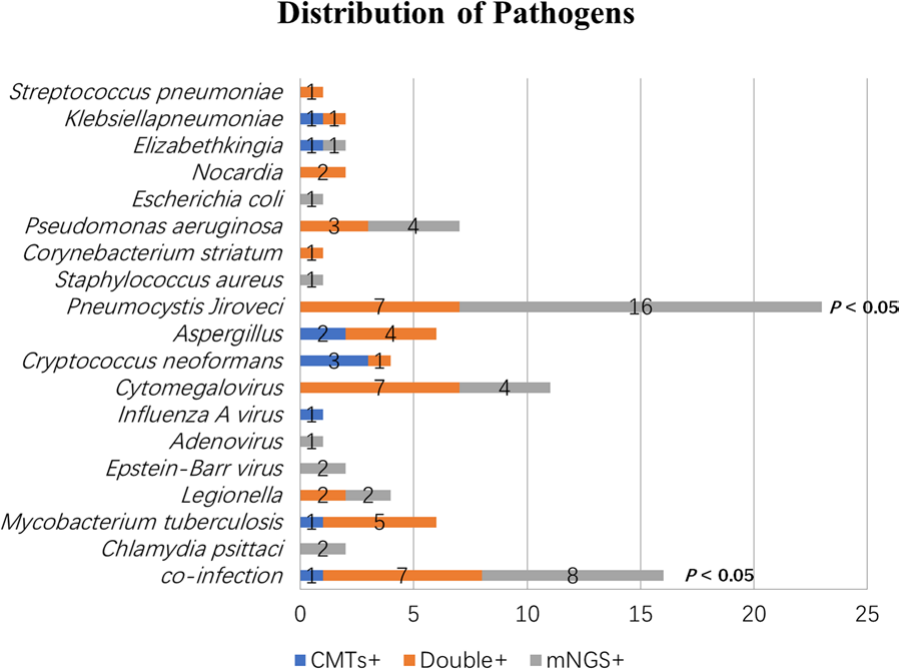
Distribution of pathogens identified in immunocompromised patients using CMTs versus mNGS. *mNGS* metagenomic next-generation sequencing; *CMTs* conventional microbiological tests

The distribution of pathogens identified by CMTs and mNGS for all 60 patients was shown in Fig. 2. CMTs and BALF mNGS were concordant for 31 of 60 (51.6%) patients (κ = 0.121; 95% CI: − 0.092 to 0.253) (Table 2).

**Table 2:** Comparison of positive results and agreement among mNGS and CMTs in immunosuppressed patients

Group	NGS-positive^a^	NGS-negative	Total no	Kappa, agreement
CMT-positive	22	3	25	0.121, 51.6%
CMT-negative	26	9	35	
Total no	48	12	60	

*mNGS* metagenomic next-generation sequencing, *CMTs* conventional microbiological tests

^a^Positive: patients with a positive microbiological diagnosis

The pathogens detected isolated by mNGS test including 16 *Pneumocystis jirovecii*, four *Cytomegalovirus*, four *Pseudomonas aeruginosa*, two *Chlamydia psittaci*, two *Legionella*, one *Escherichia coli*, one *Staphylococcus aureus* and one *Adenovirus*. For CMTs, two *Aspergillus*, three *Cryptococcus*, one *Mycobacterium tuberculosis complex* and one *Influenza A* were detected. Among the three cases of cryptococcosis detected by CMTs (PT32, PT40, PT69), the serum and BALF Cryptococcus capsular antigen tests were positive and *Cryptococcus neoformans* was identified by BALF culture in two cases. *Pneumocystis jirovecii* was identified by mNGS in PT32 and it was considerd as respiratory tract colonization at last in combination with relevant clinical data including serum BDG test and imaging. The PT40 was diagnosed as co-infection of *Cryptococcus* and *Aspergillus*, however, none of which was detected by mNGS test (Additional file 1).

### Comparison of diagnostic performance of mNGS and CMTs

With regard to bacterial detection, the diagnostic sensitivity and specificity of CMTs were 36% and 82% respectively. While, mNGS exhibited a diagnostic sensitivity of 80% and a specificity of 73%. mNGS had a higher diagnostic accuracy than CMTs but not statistically significantly different (75% vs. 65%, *P* = 0.23). A comparable diagnostic accuracy of mNGS and CMTs was also found for viral infections (*P* = 0.63). However, mNGS exhibited a higher diagnostic accuracy for fungal detection than CMTs (78% vs. 57%, *P* < 0.05), which mainly because of the high sensitivity of mNGS in patients with *Pneumocystis jirovecii* (100% vs. 28%, *P* < 0.05). GMS staining was positive for *Pneumocystis jirovecii* in 7 out of 25 patients with Pneumocystis jirovecii pneumonia (PJP), corresponding to a sensitivity of 28% (95% CI 16–28%). The poor sensitivity for the diagnosis of PJP could be improved to 88% with a combination of GMS staining and Serum (1,3)-b-D-glucan (BDG), however, the sensitivity and specificity were still lower than that of mNGS. Comparison of diagnostic performance of mNGS and CMTs for the different types of pathogens were showed in Table 3.

**Table 3:** Comparison of diagnostic performance of mNGS and CMTs in immunocompromised patients

	CMTs					mNGS				
	Sensitivity% (95% CI)	Specificity% (95% CI)	PPV% (95% CI)	NPV% (95% CI)	Accuracy% (95% CI)	Sensitivity% (95% CI)	Specificity% (95% CI)	PPV% (95% CI)	NPV% (95% CI)	Accuracy% (95% CI)
Bacterial pneumonia	36 (21–50)	82 (73–90)	53 (31–74)	69 (62–76)	65 (54–76)	80 (63–92)	73 (63–79)	63 (49–72)	87 (75–94)	75 (63–84)
Fungal pneumonia	50 (37–62)	63 (50–75)	57 (42–71)	54 (45–67)	57 (44–69)	91 (79–98)	66 (54–72)	72 (63–77)	89 (73–97)	78 (66–85)
*Pneumocystis jirovecii*^a^	28 (16–28)	100 (91–100)	100 (58–100)	71 (66–71)	74 (66–74)	100 (86–100)	86 (79–86)	81 (70–81)	100 (90–100)	91 (82–91)
*Pneumocystis jirovecii*^b^	88 (72–97)	84 (75–89)	76 (62–83)	93 (83–98)	86 (74–92)	100 (86–100)	86 (79–86)	81 (70–81)	100 (90–100)	91 (82–91)
*Aspergillus* spp.	86 (63–99)	97 (92–98)	75 (41–87)	98 (94–100)	96 (88–98)	57 (23–71)	98 (95–100)	80 (33–99)	95 (92–97)	94 (87–97)
Viral pneumonia	53 (34–53)	100 (95–100)	100 (64–100)	89 (84–89)	90 (81–90)	93 (70–100)	83 (77–85)	61 (46–65)	98 (90–100)	86 (75–88)

*mNGS* metagenomic next-generation sequencing, *CMTs* conventional microbiological tests, *PPV* positive predictive value, *NPV* negative predictive value

^a^Detect by Gomori methenamine silver staining only

^b^Detect by a combination of Gomori methenamine silver staining and serum (1,3)-b-D-glucan (≥ 80 ng/l was defined as positive)

### Comparison of mNGS and CMTs in co-infection

**Fig. 3:**
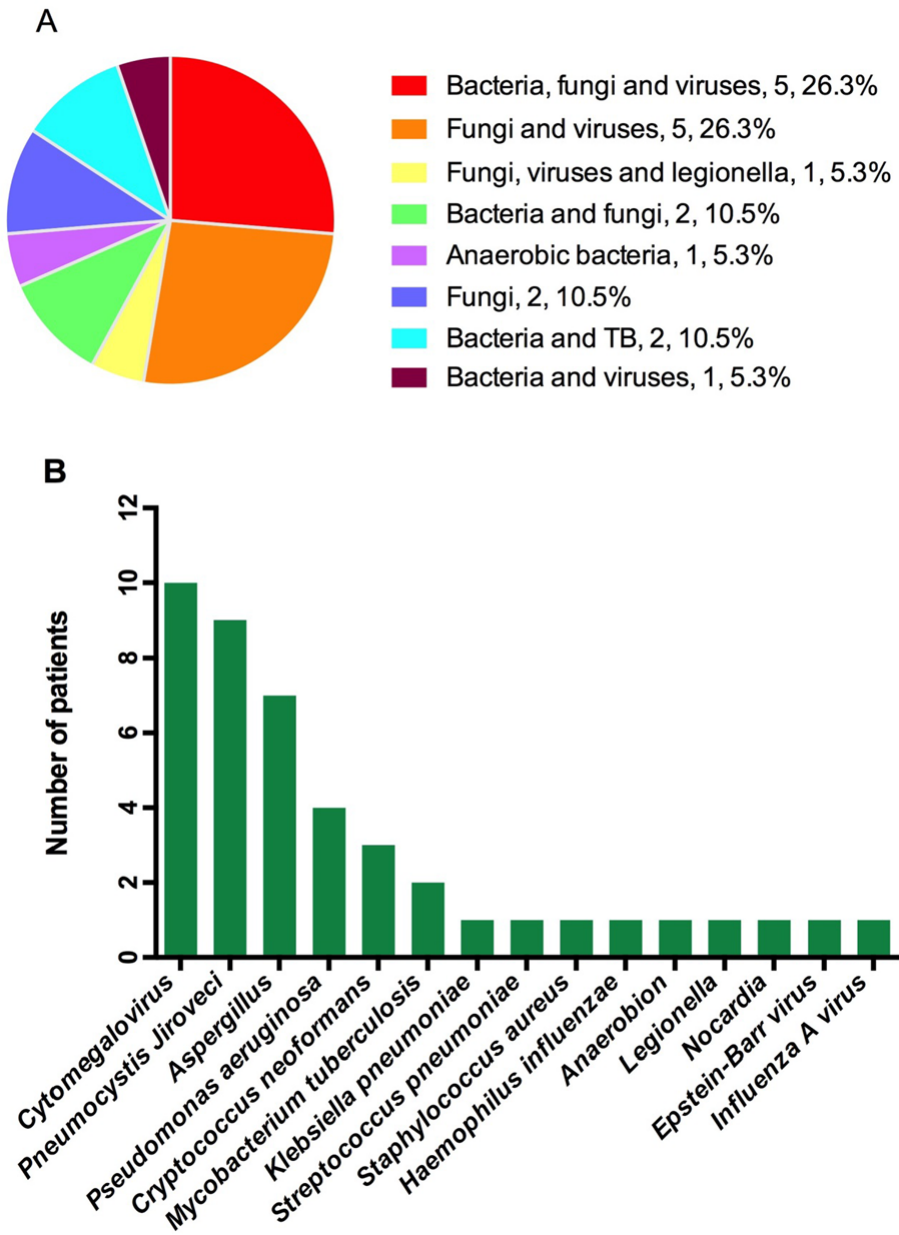
Distribution of pathogens identified in immunocompromised patients with co-infection. **A** Percentage of patients with co-infection for various pathogens. **B** Pathogen spectrum of immunocompromised patients with co-infection. *TB* Mycobacterium tuberculosis

19 patients were diagnosed with co-infection (including nine cases with two pathogens, nine cases with three pathogens and one case with more than three pathogens). The detection rate of mNGS for co-infection was significantly higher than that of CMTs (79% vs. 42%, *P* < 0.05) and more co-pathogens could be identified by mNGS. Fungi-virus-bacteria and fungi-virus coinfection were the most common co-pathogens observed in co-infection patients. The most common pathogen in co-infection was *Pneumocystis jirovecii* (9/19, 47.4%) and *Cytomegalovirus* (10/19, 52.6%) (Fig. 3 and Additional file 2).

## Discussion

Although many researches have evaluated the performance of mNGS in different patients of different infection types [11, 17], there is still lack of studies on the utility of mNGS of BALF in immunocompromised hosts with pneumonia. In a multicenter prospective study of 329 severe community-acquired pneumonia (SCAP) patients, a higher detection rate (up to 90.3%) and an earlier detection for pathogens by mNGS of BALF than that of CMTs was reported [7]. However, immunocompromised cases represented less than 20% of the patients enrolled. On the contrary, Peng et al. showed a similar diagnostic performance of mNGS of BALF to CMTs for all types of pathogens in 60 immunocompromised patients [6]. In this study, a higher microbial detection rate and shorter time requirement for pathogen identification were showed in immunocompromised patients for using mNGS test of BALF than that of CMTs. Unlike Peng’ study, we have found an improved diagnostic accuracy of BALF mNGS compared with that of CMTs in fungal infections, which may be due to the high-proportion of pneumocystis and low-proportion of aspergillus in this study, and the diagnostic criteria we used (genome coverage information) may have more advantage than Peng’s (SMRN (the stringently mapped read number) in species level) for identify fungi infection when the detected reads number is low.

*Pneumocystis jirovecii* was the most common opportunistic pathogen in immunocompromised patients [18]. It was reported to be responsible for 61.2% of confirmed pneumonia in immunocompromised patients [6]. Compare to CMTs, mNGS showed remarked advantages for detecting *Pneumocystis jirovecii*. Wang et al. [19] showed significantly higher sensitivity of mNGS test than GMS alone for diagnosis PjP (100% vs. 30.7%). In this study, the poor sensitivity of GMS for diagnosis PJP was remarkably improved with the addition of serum BDG test. However, it was still lower than that of mNGS. These results suggest that mNGS is a useful diagnostic tool with good performance for the diagnosis of PJP.

More polymicrobial infections were found in immunocompromised patients in comparison with immunocompetent patients [20]. Pathogen detected by CMTs methods is usually a single one, while mNGS can detect multiple pathogens at the same time because of its unbiased detection technology, which has more advantages in the diagnosis of co-infection [19, 21]. In this study, mNGS showed a higher detection rate and broader spectrum for pathogen detection than that of CMTs in co-infection. Moreover, *Pneumocystis jirovecii* was found to be the most common pathogen in pulmonary co-infection, which was similar to the previous study [22]. Compare to CMTs, mNGS have identified much more co-pathogens of PJP. These results indicated that a remarkable advantage of mNGS method for identify mix pulmonary infection and detect co-pathogens of PJP than CMTs.

However, it was still a challenge for mNGS to identify *Aspergillus* or *Cryptococcus* because of the difficulty of DNA extraction from the thick polysaccharide cell wall [13, 23]. In our study, the diagnosis sensitivity for IPA by CMTs is a litter higher than that of mNGS. As for cryptococcosis, the sensitivity arrived 100% by using CMTs, while only one case was identified by mNGS.

Our retrospective study contains certain limitations. Firstly, as a retrospective study, there were selection bias and recall bias which was inevitable. In addition, the lack of RNA sequencing and partial of PCR methods had failed to evaluate the diagnostic value of CMTs and mNGS better especially for virus infection. The interpretation of virus detected by mNGS was depended on the clinician's subjective judgment more than diagnostically confirmed. Finally, it was difficult to distinguish pathogens from colonization to infection due to the unbiased detection of mNGS without unified standard. However, the emergence of real-time metagenomics, as described by Morsli et al. [24], which can reduce contamination rates and provide pathogen genomic surveillance in real time for diagnosis, genotyping and bacterial profiling may resolve the limitations encountered in our study in the future. In addition, future prospective studies should pay more attention to develop databases containing the pathogens involved in this disease for more accuracy in the mNGS findings and clinical interpretation and improve the mNGS protocols including sampling, nucleic acid extraction, human genome and/or biofilm depletion, and a porpose of additional microbial enrichment step in library preparation protocol.

## Conclusion

mNGS using BALF improved the microbial detection rate of pathogens espically in the case of limited CMTs and exhibited remarkable advantages in detecting PJP and identifying co-infection in immunocompromised patients. However, it is still a challenge for mNGS using BALF to detect *Aspergillus* or *Cryptococcus* infection.

## Supplementary Information

**Additional file 1.** Original data.

**Additional file 2.** Identified co-infection by CMT and mNGS.

## Data Availability

The datasets generated and/or analysed during the current study are available in the National Center Biotechnology Information BioProject database under accession number PRJNA808886.
